# Analysis of Determinant Factors and Mechanisms in Early Childhood Care Services: A Qualitative Study in the Asturian Context (Spain)

**DOI:** 10.3390/children12081079

**Published:** 2025-08-17

**Authors:** Yara Casáis-Suárez, José Antonio Llosa, Sara Menéndez-Espina, Alba Fernández-Méndez, José Antonio Prieto-Saborit, Estíbaliz Jiménez-Arberas

**Affiliations:** Faculty Padre Ossó, University of Oviedo, 33008 Oviedo, Spain; yaracasais@facultadpadreosso.es (Y.C.-S.); joseallosa@facultadpadreosso.es (J.A.L.); saramenendez@facultadpadreosso.es (S.M.-E.); albafernandez@facultadpadreosso.es (A.F.-M.); estibaliz@facultadpadreosso.es (E.J.-A.)

**Keywords:** early childhood care, context, care system

## Abstract

**Highlights:**

**What are the main findings?**

- System improvement from a comprehensive perspective: Our findings suggest the need to address structural limitations—organizational, professional, and logistical—within the early intervention system.
- Enhancement of intersectoral coordination: Collaboration needs to be fostered between healthcare, education, and social services, which is essential for more effective and cohesive care.

**What is the implication of the main finding?**

- Development of rural-context-sensitive policies: Early childhood intervention services must be adapted to the geographical, demographic, and social specificities of rural areas in Asturias.
- Strengthening territorial equity: Resources must be distributed and targeted strategies designed to reduce access inequalities between urban and rural areas.

**Abstract:**

Diverse realities challenge the management capacity of public and private systems to ensure equitable quality and efficient access to resources, in line with the 2030 Agenda and the Sustainable Development Goals, which aim to close gaps in essential services and ensure quality of life. The reality in Spain, and more specifically in the Principality of Asturias, is that most resources are concentrated in urban areas rather than rural ones, partly due to the region’s geography. **Background/Objectives**: This study aimed to explore the perspectives of various stakeholders on the early childhood care system in the Principality of Asturias (Spain), with the purpose of analyzing the mechanisms and determinants involved in its functioning and identifying opportunities for improvement. **Methods**: A qualitative study was conducted using the theoretical framework of the National Institute on Minority Health and Health Disparities (NIMHD) as a conceptual basis. Semi-structured interviews were carried out with 24 participants selected based on their relationship with early childhood care systems, encompassing different levels of responsibility and operational roles. Data were analyzed using a phenomenological approach, employing inductive and deductive coding to identify recurring patterns and code co-occurrences within ATLAS.ti software. **Conclusions**: This study reveals major barriers to equitable early childhood intervention (ECI) in rural areas, such as geographic isolation, lack of specialists, long waiting times, and poor transport. Six key themes emerged, including the need for standardized system management, better family support, and digital tools like centralized electronic health records. Rural areas are directly limited regarding their access to services, highlighting the need for fair territorial planning and a holistic, inclusive care model. Improving coordination, accessibility, and technology is vital.

## 1. Introduction

Early childhood care is founded on the principle of offering children with disabilities the opportunity to participate in their community as full and equal members [1,2]. To achieve this goal, the care system facilitates therapeutic interventions aimed at enhancing children’s developmental abilities, improving their functionality, and promoting their participation in diverse contexts and daily activities [3,4,5,6].

The essential framework of care systems is rooted in theoretical health models, which consider numerous constructs that influence, to varying degrees, the implementation and effectiveness of strategies that foster inclusion and holistic well-being for children and their families [7,8]. The implementation of these models was affected by the health crisis caused by COVID-19, which exposed weaknesses in perinatal and early childhood care systems. These included key issues such as disruptions in continuity of care, a lack of intersectoral coordination, and an increasing emotional burden on families [9]. In Spain, a substantial share of children with neurodevelopmental disorders (NDDs) remain undiagnosed; up to 20% of children present with any NDD, amounting to hundreds of thousands of school-aged children. Delayed detection forfeits critical windows of neuroplasticity and increases the risk of functional disability, academic failure, family stress, and long-term societal costs [10].

While these theoretical models provide a comprehensive perspective on health and shared objectives, the availability and operation of services vary by region and local circumstances, making practical implementation a significant challenge. In rural areas, residents face a range of obstacles stemming from the inherent characteristics of these settings [11,12,13], particularly for children with early childhood intervention needs. Among the most prominent barriers are the limited access to reliable internet connections, unlimited data plans, and the necessary electronic devices to engage with telehealth services effectively. This technological gap often hinders the ability of rural populations to benefit from remote care options, further exacerbating healthcare access issues [14].

In addition, rural residents frequently encounter long wait times and must undertake extensive travel to access therapeutic services, which is a situation that poses additional logistical and financial burdens for families. These barriers are particularly pronounced in areas where resources beyond the early care stage are scarce, leaving individuals with limited options for continued care and support [15]. Furthermore, rural regions often face a shortage of specialized healthcare professionals, coupled with challenges in retaining them, especially in areas that are distant from urban population centers.

These factors have been widely recognized in the literature as key contributors to the persistence of health inequalities in rural communities. The combination of limited resources, long travel times, and difficulties in accessing specialized care serves to reinforce existing health disparities, making it crucial to address these issues in order to improve healthcare equity in underserved areas [11,14].

This diversity of realities challenges the capacity of systems and organizations to meet the criteria of equity, quality, and efficiency, as outlined in the 2030 Agenda and the Sustainable Development Goals [16], which aim to close gaps in access to essential services and ensure quality of life for all individuals.

This study aims to explore the perspectives of various stakeholders on the early childhood care system in the Principality of Asturias (Spain), analyzing the mechanisms and determinants involved in its operation. Specifically, the objective was to conduct an in-depth analysis of the characteristics of the population served by early childhood care services, the system’s capacity to address the needs of this population group, the ability of stakeholders at various levels to coordinate effectively, and, finally, the potential improvements that could be implemented.

## 2. Materials and Methods

### 2.1. Theoretical Framework

For this study, the Research Framework of the National Institute on Minority Health and Health Disparities (NIMHD) (Bethesda, MD, USA) [17] was selected as the foundation for exploring variations in how families and early childhood care providers perceive the delivery of care services.

This framework is commonly adopted in qualitative studies aiming to examine the multidimensionality of health in marginalized communities. The present study considers rurality as a factor that may influence quality-of-care services, potentially leading to differences among members of the population.

### 2.2. Data Collection and Analysis

The sampling of participants was structured based on different levels of involvement in early childhood care to obtain a comprehensive and holistic understanding of the system. Given the qualitative design and the hard-to-reach nature of the target population, we employed a non-probability snowball sampling strategy. Initial “seed” participants (n = 13) were identified purposively. This included various degrees of responsibility and operational roles, as well as diverse levels of abstraction in the narratives of those providing and receiving services. A total of n = 27 participants were included in the study and were organized according to their relationship with early childhood care services in the Principality of Asturias, a region located in the north of Spain. Demographic aspects are presented in Table 1.

Data were collected through semi-structured interviews, where participants were asked to share their perceptions and experiences related to four thematic axes: population characteristics, the system’s capacity for care, coordination capacity, and proposals for improvement. The interview guide was designed to describe, from an academic and professional-practice perspective, the state of early childhood intervention in the Principality of Asturias (Spain), from the characteristics of the population served. The semi-structured interview comprised 15 open-ended questions distributed across four thematic axes: Axis 1 (population characteristics), 4 questions; Axis 2 (system care capacity), 7 questions; Axis 3 (coordination capacity), 1 question; and Axis 4 (proposals for improvement), 3 questions. The guide was also organized into four thematic axes with open-ended questions and probing sub-questions that allowed a deeper exploration according to the informant’s experience: (1) Axis 1. Population characteristics (Asturian context: the most frequent case profiles in AIT and elements that differentiate Asturias from other autonomous communities) and rural vs. urban differences in detection opportunities, access, and service provision by territory. (2) Axis 2. System care capacity (entry and exit mechanisms: understanding how the AIT system operates in Asturias; age coverage (up to 3 years) and the assessment of a possible extension to 6 years; adequacy of post-discharge pathways and the existence/quality of the transition to early childhood education) and professional profiles (their adequacy, underuse, or need to reconfigure the profiles of those present/absent in services). (3) Axis 3. Coordination capacity (interprofessional and intersectoral coordination). (4) Axis 4. Proposals for improvement (the level of care provided and intersectoral and interprofessional coordination). The interview was intended to be conducted individually in person (n = 7) or online (n = 20), with an estimated duration of 45–60 min; an audio recording was taken following informed consent, and subsequent verbatim transcription. The semi-structured nature of these interviews ensured comparability across interviews (shared axes and core questions) while allowing flexibility to explore emerging dimensions in depth. The axes and questions were derived from the AIT literature, national and regional regulatory frameworks, and current academic debates. The guide was reviewed by the research team (and, where applicable, by external experts) to optimize clarity, relevance, and thematic coverage, and piloted in an initial interview to adjust ordering, phrasing, and probes.

This study was approved by the Bioethics Committee of the Principality of Asturias with code CEImPA 2023.342, in compliance with Organic Law 3/2018 of December 5 on Data Protection and the Guarantee of Digital Rights.

Data analysis was conducted using a phenomenological approach through thematic analysis. The software ATLAS.ti Mac (version 24.2.0) was utilized for coding and identifying recurring themes and patterns. A mixed methodology of inductive and deductive coding was employed across three phases [18]: identifying units of meaning, assigning codes, and creating categories.

An analysis of code frequency and co-occurrence was performed to identify factors that provide a comprehensive view of the early childhood care system. Finally, thematic analysis was conducted based on the identified categories to corroborate the associations between frequency and co-occurrence results and the content of the interviews.

### 2.3. Evaluation of Study Quality

To ensure rigor in this research, the Consolidated Criteria for Reporting Qualitative Research (COREQ) [19] and the JBI Critical Appraisal Checklist for Reporting Qualitative Research [18] were applied to ensure theoretical and methodological consistency of the results. See Appendix B for further details.

## 3. Results

**Figure 1:**
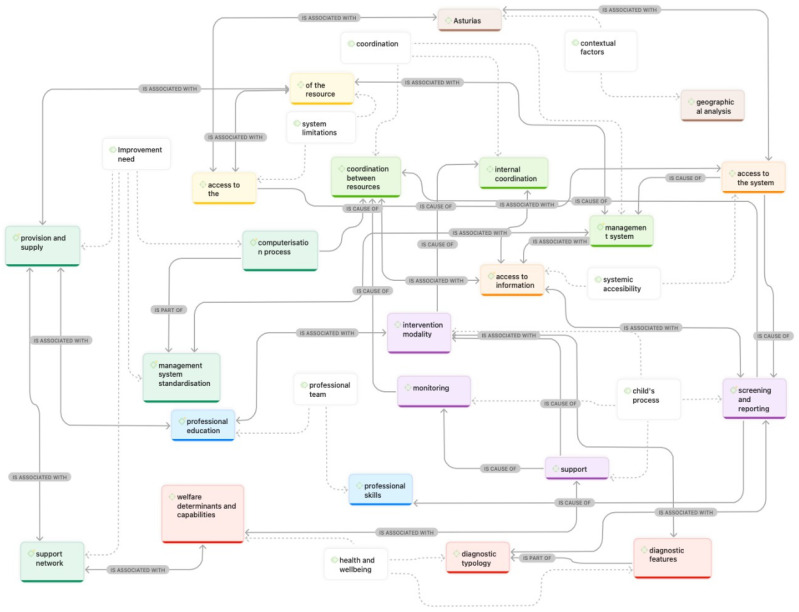
Network view of relationships between codes and code families.

This research provides a comprehensive view of the early childhood care system in the Asturian context, identifying recurring themes emerging from various perspectives. A total of 143 codes were created and subsequently grouped into eight categories (Table 2). Codes not associated with any category were eliminated, using frequency of occurrence as an indicator of empirical relevance [20] and after confirming their theoretical irrelevance [21]. Figure 1 shows the aggrupation’s code and its absolute and relative frequency according to the discourse level. Since the number and length of interviews varied depending on the discourse level, normalized values were used to reduce bias and provide a more accurate analysis [21,22,23].

Figure 1 presents a view of the network, illustrating the interconnection between codes and groups of codes, indicating that certain themes associated with the early childhood care system in the Principality of Asturias coexist across different discourse levels.

The results suggest that the group of codes related to coordination is linked to systemic accessibility and the child’s journey through care services, which impacts health and well-being. Additionally, it is observed that the code “Asturias” is associated with the system’s coordination and its limitations. This indicates that context and environment are key factors when making decisions about the functioning and quality of the service.

A significant indicator of the relationship between different themes is provided through code co-occurrence, which represents 10–20% of the total appearances of a code [20]. See the Supplementary Materials for the figure that presents the co-occurrence analysis and the significance between codes.

Considering the highest co-occurrence coefficients, the results suggest a close relationship between three pairs of codes (see the Supplementary Materials). On the one hand, the code “access to information” co-occurs with the code “management system” on 19 occasions (*p* = 0.14), suggesting a link between access to information and decision-making regarding the structuring of the system. Meanwhile, the code “Asturias,” which refers to the particularities of the regional context, co-occurred 20 times (*p* = 0.21) with the code “access to the resource,” which highlights both physical (e.g., transportation) and systemic limitations (e.g., waiting lists) in ensuring children receive care. This co-occurrence indicates that accessibility issues may be influenced by the specific characteristics of the Asturian context. Finally, the codes “management system” and “management system standardization” which refer to coordination and improvement needs, respectively, co-occurred 17 times (*p* = 0.14).

### Thematic Analysis of Categories

Several key themes were identified based on the functioning of the early childhood care system, highlighting its strengths and limitations. Appendix A presents the most representative quotes from the different themes extracted.

Theme 1: Systemic Accessibility

Systemic accessibility emerged recurrently in interviews, particularly among families who reported significant difficulties in accessing information and care resources. The co-occurrence between the codes “access to information” and “management system” (*p* = 0.14) highlights, on the one hand, the importance of providing high-quality, continuous information to beneficiaries about the system and their own care process, and on the other hand, the need for effective communication channels among professionals to optimize processes.

Theme 2: Coordination

The early childhood care management system was discussed more extensively by policymakers and managers than at other discourse levels. The co-occurrence coefficient between the codes “management system” and “management system standardization” (*p* = 0.14) underscores the importance of establishing protocols to enable greater fluidity in intersectoral coordination. Academics supported this notion, adding that the lack of standardized circuits affects both the internal management of processes and the quality of care provided.

Theme 3: System Limitations

System limitations emerged as a recurring theme across all discourse levels, particularly regarding access to resources. Notably, at the political level, references to resource limitations were absent, suggesting that the system’s challenges at a micro level are predominantly perceived by technical staff. The co-occurrence between the codes “Asturias” and “access to the resource” (*p* = 0.21) reflects the difficulties faced by families, particularly in rural areas, due to the characteristics of the local environment.

Additionally, the pairing of the codes “intervention modality” and “provision and supply” (*p* = 0.09) appears to indicate the need for greater resources to ensure the adequate support and care of children in this context.

Theme 4: Contextual Factors

Variables related to contextual factors emerged with notable frequency across the different discourse levels. Although co-occurrences do not reflect a particularly strong association, this theme could be considered a determinant for the effective development of others. Among the defining characteristics of the Asturian context were a dispersed population distribution, low birth rates, a higher prevalence of language delays, autism spectrum disorder cases, and limited access to services for rural families. These factors strongly influence service provision.

Theme 5: Improvement Needs

The co-occurrence between the codes “management system standardization” and “management system” underscores the need for systematic processes that can identify and refer children to the early childhood care system. Similarly, the pairing of the codes “computerization process” and “management system” also co-occurred significantly (*p* = 0.10), suggesting the necessity of creating a unified clinical record to monitor children’s progress throughout their journey in the system. This would facilitate the flow of information among professionals within the sector.

Theme 6: The Child’s Process Within the System

This theme includes aspects related to assessment, intervention modalities, and child follow-up, all of which are crucial for fostering child development. As noted, the inter-institutional convergence inherent in the mechanisms for childcare is highly complex and aims to achieve the highest standards of family well-being.

Participants highlighted the importance of early detection, enabling continuous and individualized interventions throughout the child’s journey within the system (*p* = 0.12 between the codes “access to the system” and “screening and reporting”). Additionally, in terms of intervention, the need to establish a support network for families was emphasized to ensure they feel supported and secure in their decision-making. This idea is reflected in the co-occurrence between codes “support” and “intervention modality” (*p* = 0.12).

## 4. Discussion

This study aimed to explore the perspectives of key stakeholders regarding the early childhood care system in the Principality of Asturias (Spain), with the goal of analyzing the mechanisms and determinants that influence its operation. The findings of this research reveal several significant challenges that affect three fundamental aspects of the care process: access to and delivery of services, family involvement in the care process, and coordination efforts among professionals in areas removed from urban centers. These results are consistent with previous research emphasizing the importance of ensuring equitable access to services and involving families in decision-making processes to optimize the outcomes of interventions [24,25].

One of the main challenges identified in this study was access to services in rural areas of the Principality of Asturias, both in terms of physical barriers (such as transportation) and systemic obstacles (such as waiting lists), which limit children’s ability to receive timely care. Both professionals and families reported that geographical constraints undoubtedly hinder access to specialized resources. This finding aligns with a study conducted in China, which identified three primary reasons for the lack of healthcare services in rural areas: the concentration of service demand in more densely populated zones, which leads to the centralization of services and allocation of more resources to families residing in urban environments, often undermining the principle of equity in care delivery; the shortage of professionals, driven by higher demand and better infrastructure in central areas, making it difficult to ensure professional coverage in rural zones, especially given the unsatisfactory compensation, limited opportunities for professional development, and poor living conditions; and, finally, the transportation network, travel time, and fuel costs, all of which were also highlighted by various stakeholders in our study as major barriers to accessing early childhood intervention services [26,27].

Another concern raised by the participants was related to waiting times for accessing early intervention services, which influence both the timeliness of diagnosis and the overall duration and quality of the care received. This systemic limitation in responding to the needs of children and families was also evidenced in the study by Barnard-Brak, Morales-Alemán, Tomeny, and McWilliam (2021) [15].

In summary, geographical dispersion and the centralization of resources in urban centers limit the reach of services in peripheral areas, thereby impacting the equity of the system [11,12,28]. This phenomenon is not unique to the Principality of Asturias; international studies have identified similar barriers in other health and early childhood development systems [11,15,29].

With regard to family participation, it is considered essential to allocate resources in ways that are responsive to the needs and interests of families. Manjarrés-Carrizalez and León-González (2024) [30] provided data on the relationship between raising a child with disabilities and a decrease in caregivers’ quality of life. Their involvement in decision-making processes and continuous access to information about the care process have a positive impact on child development [25], correlating with higher levels of functionality and well-being in the home environment. The adoption of family-centered care models by professionals aligns with this perspective and further emphasizes the need for qualified personnel capable of transferring knowledge and providing individualized support to families [31], thereby ensuring the generalization of learning for both parties in natural environments.

Another key issue identified in this study was professional coordination. Participants noted that the lack of time and human resources hampers adequate coordination between different services, resulting in fragmented interventions. This issue has already been documented in the literature and underscores the importance of establishing coordinated teams to ensure the effective implementation of early childhood interventions [32,33].

In terms of improvement strategies, this study’s findings suggest that process digitalization could serve as a key solution to enhancing efficiency and coordination among professionals. Some studies report that technology is the primary communication tool among professionals. As families and therapists become more familiar with technology and the capacity of communication networks increases in rural and remote areas, the potential for early childhood professionals to use technology in innovative ways to provide guidance and support to geographically isolated families is maximized. Furthermore, another improvement measure proposed by stakeholders at various levels in our study is the implementation of a centralized digital health record. Such a system would allow professionals to access children’s information more quickly and efficiently, reduce intervention fragmentation, improve internal communication, and unify the information provided to families [34].

This study has several limitations that should be considered when interpreting its findings. First, it is context-bound to the Principality of Asturias, a territory with a highly specific rural–urban configuration (e.g., population dispersion, geographic barriers, and sociodemographic particularities). Consequently, the transferability of the results to other autonomous communities—or to more densely populated or differently organized rural regions—may be limited. Even though some of these territorial features align with variables examined in prior research on the effects of rurality, further studies in comparable settings are warranted to examine the consistency of these patterns. Second, the qualitative methodology privileges depth over breadth and captures the perspectives of the interviewed stakeholders, which are necessarily shaped by their roles, experiences, and positionalities. This raises issues of representativeness, selection bias, social desirability, and the potential under-representation of certain voices (e.g., families from low socioeconomic backgrounds and migrant or minority groups). Finally, this study’s focus on professional/academic/family perspectives may limit insights into the longitudinal evolution of care pathways and the direct experiences of children and their families. Future work should adopt mixed-methods, longitudinal or comparative multi-region designs, and participatory approaches to better capture the complexity of early childhood intervention in rural contexts and inform scalable, equity-oriented policy solutions.

## 5. Conclusions

This study highlights the significant structural barriers affecting access to and the quality of early childhood intervention services in rural areas of the Principality of Asturias. Geographic dispersion, a shortage of specialized professionals, insufficient public transportation, and long waiting times all hinder equitable service delivery and negatively impact both child development and family well-being.

These findings underscore the need for more equitable territorial planning, including adequate allocation of human and material resources in rural contexts, as well as improved working conditions to attract and retain professionals in these settings. Additionally, key areas for improvement include enhanced interprofessional coordination and system digitalization, particularly the implementation of a centralized digital health record to support continuity of care.

Ensuring fair and context-sensitive access to ECI services is essential for advancing toward a more inclusive and effective care model.

## Figures and Tables

**Table 1:** Participant demographics (n = 27).

	N (%)
Gender	
Male Female	4 (14.8) 23 (85.2)
Discourse level	
Politician or executive Technician Academic Families	4 (14.29) 17 (60.71) 4 (14.29) 2 (7.14)

**Table 2:** Code groups by discourse levels.

	Academics	Families	Politicians and Experts	Technicians	Total
Intervention modality	11.164 (16.2%)	20.991 (30.45%)	15.775 (22.89%)	21.00 (30.47%)	68.93
Monitoring	1.861 (9.7%)	5.248 (27.37%)	6.067 (31.64%)	6.00 (31.29%)	19.176
Screening and reporting	1.861 (6.28%)	15.744 (53.18%)		12.00 (40.53%)	29.604
Support	5.582 (9.70%)	34.111 (59.28%)	4.854 (8.43%)	13.00 (22.59%)	57.547
Asturias	27.909 (30.11%)	18.368 (19.82%)	19.415 (20.95%)	27.00 (29.13%)	92.692
Geographical analysis	16.745 (56.67%)		4.854 (16.40%)	8.00 (27.03%)	29.599
Coordination between resources	13.024 (24.36%)	7.872 (14.73%)	14.561 (27.24%)	18.00 (33.67%)	53.457
Internal coordination	5.582 (22.98%)	10.496 (43.21%)	1.213 (5.00%)	7.00 (28.82%)	24.291
Management system	26.048 (22.17%)	10.496 (8.93%)	50.964 (43.37%)	30.00 (25.53%)	117.509
Diagnostic features		18.368 (93.80%)	1.213 (6.20%)		19.581
Diagnostic Typology	9.303 (23.20%)	18.368 (45.81%)	2.427 (6.05%)	10.00 (24.94%)	40.097
Welfare determinants and capabilities	13.024 (23.81%)	13.12 (23.98%)	14.561 (26.62%)	14.00 (25.59%)	54.705
Computerization process	9.303 (30.93%)		15.775 (52.45%)	5.00 (16.62%)	30.078
Management system standardization	18.606 (29.26%)		33.976 (53.44%)	11.00 (17.30%)	63.582
Provision and supply	46.516 (44.96%)	5.248 (5.07%)	26.696 (25.80%)	25.00 (24.16%)	103.459
Support network	13.024 (35.97%)	2.624 (7.25%)	14.561 (40.21%)	6.00 (16.57%)	36.209
Professional education	1.861 (13.71%)		9.708 (71.55%)	2.00 (14.74%)	13.568
Professional skills	14.885 (27.18%)	5.248 (9.58%)	20.628 (37.67%)	14.00 (25.57%)	54.761
Access to the resource	18.606 (23.08%)	28.863 (35.81%)	12.134 (15.05%)	21.00 (26.05%)	80.604
Limitations of the resource	13.024 (32.44%)	13.12 (32.68%)		14.00 (34.87%)	40.144
Access to information	11.164 (9.80%)	57.726 (50.66%)	23.055 (20.23%)	22.00 (19.31%)	113.945
Access to the system	27.909 (33.04%)	20.991 (24.85%)	14.561 (17.24%)	21.00 (24.86%)	84.462
Total	307.00 (25%)	307.00 (25%)	307.00 (25%)	307.00 (25%)	1228 (100%)

## Data Availability

The original contributions presented in this study are included in the article and Supplementary Materials. Further inquiries can be directed to the corresponding author.

## Supplementary Materials

The following supporting information can be downloaded at: https://www.mdpi.com/article/10.3390/children12081079/s1, Figure S1: Co-occurrence of codes in the qualitative analysis of the study.

## Abbreviations

COREQ	Consolidated Criteria for Reporting Qualitative Research
ECI	Early Childhood Intervention
NDDs	Neurodevelopmental Disorders
NIMHD	National Institute on Minority Health and Health Disparities

## Appendix A

**Themes**	**Representative Quotes**
Theme 1: Systemic Accessibility	**Family 1:** “[…] I didn’t receive any information, they just say ‘today went well,’ but that’s all I know.” **Family 2:** “[…] The report is given to you at the very last moment, so it doesn’t matter whether you request it or not, because they must meet to prepare the report. The professional (from early care centre) might tell me verbally that things are improving or whatever, but nothing more.” **Technician 8:** “[…] Communication, in my experience, whether it’s with education, early care, or anyone, not even with XXXX, who is right next door, it needs significant improvement. […] We communicate exclusively through reports.”
Theme 2: Coordination	**Academic 3:** “[…] They are not interconnected vessels (each medical speciality), they are separate worlds. So, we all communicate through medical reports. Some reports are good and keep you informed, and others are less so, leaving you less informed. But there is no systematic approach in place.” **Academic 3:** “It would be fantastic to have at least a minimal medical record system so that people wouldn’t have to keep carrying papers back and forth.” **Politician 1:** “Yes, coordination… especially between health and social services, […] I am aware that there are limitations in the systems for information exchange. We are currently developing the unified electronic social record.”
Theme 3: System Limitations Theme 4: Contextual Factors	**Family 2:** “[…] Honestly, it’s been very difficult to travel from here to there. Besides being far, the road is winding, and when I go to Arriondas, I end up feeling unwell, and even the baby has also arrived feeling unwell.” **Family 2:** “[…] The bus leaves at a certain time, we get there, and she (the early care professional) gives her the session, then we come out and take the bus back again… Honestly, it’s very difficult to… to make the trip.” **Technician 12:** “[…] The waiting lists are generated there (in the regional centres), where we have the highest demand and where there is a lack of adequate care […] We cannot provide the sessions that each child needs because our services are overwhelmed.” **Academic 3:** “[…] They are often diagnosed late […], they arrive late and then the care they receive from Public Services, is only what can be provided because the system is overwhelmed.” **Academic 2:** “[…] A parent and a child who must be taken to the (urban) centre for evaluation and other things, just think about the number of trips they must make for follow-ups or interventions. If everything is done correctly to avoid wasting time […] and well… you already know how long the waiting times are.”
Theme 5: Improvement Needs	**Politician 1:** “Yes, coordination… especially between health and social services, […] I am aware that there are limitations in the systems for information exchange. We are currently developing the unified electronic social record.” **Technician 11:** “Early Childhood Care and education, I believe, are complementary rather than mutually exclusive. And I think… they could coexist.” **Technician 11:** “Regarding the age of the children, I think we should provide care until they are six. For that, we would need a broader network of professionals and larger Early Care Centres to make it possible. […] We must care for all of them… It’s not acceptable to say they will be cared for until six but have a waiting list”.
Theme 6: The Child’s Process Within the System	**Technician 5:** “[…] What I’ve been noticing over these 12 years working here in the unit is that detection is becoming increasingly earlier due to the growing knowledge available.” **Technician 13:** “[…] Detection can also be carried out by the family itself […] They notice or think there might be some kind of difficulty, they go to the paediatrician, and it’s the paediatrician who refers them to health services.” **Technician 13:** “[…] In early care sessions, the objectives that the child needs are addressed, tackling them from different areas. If they need more language support, it’s handled through speech therapy; if it’s more cognitive, through pedagogy or psychology.”

## Appendix B

### Appendix B.1. Consolidated Criteria for Reporting Qualitative Research (COREQ)

**ITEM**	**Question/Description**	**Response**
Domain 1: Research team and reflexivity		
Personal characteristics		
Interviewer/facilitator	Which author(s) conducted the interviews or focus groups?	All authors participated in at least one interview.
Credentials	What were the researcher’s credentials?	PhDs with extensive experience in interviews, qualitative methodology, and childhood.
Occupation	What was the occupation of the authors during the study?	Researchers.
Gender	Was the researcher male or female?	Female.
Experience and training	What experience or training did the researcher have?	Specific training and prior experience in qualitative studies using focus groups/interviews.
Relationship with participants		
Relationship established	Was a relationship established prior to the study?	No.
Participant knowledge of the interviewer	What did the participants know about the interviewer?	Participants were not given formal information about the researcher beforehand. However, at the start of the interview, details of their training, credentials, and position were provided.
Interviewer characteristics	What characteristics were reported about the interviewer/facilitator?	
Domain 2: Study design		
Theoretical framework		
Methodological orientation and theory	What methodological orientation was applied to support the study?	A qualitative approach based on phenomenology and thematic analysis was used.
Participant selection		
Sampling	How was the sample selected?	Sampling was based on the level of involvement in the early childhood care system. Convenience sampling identified key informants.
Method of interviews	How were participants interviewed?	In person or online.
Sample size	How many participants were included in the study?	N = 28.
Non-participation	How many participants refused or dropped out?	N = 0.
Setting		
Interview location	Where was the data collected?	Researchers traveled to the interviewee’s preferred location for face-to-face interviews.
Presence of non-participants	Was anyone else present besides the interviewer and participants?	No.
Sample description	What are the fundamental characteristics of the sample?	The sample included families, technical staff/coordinators, academics, and policymakers.
Data collection		
Interview guide	Were questions, prompts, or guides provided by the authors? Were they tested?	A shared interview guide based on four thematic axes was adapted to each sample group.
Repeat interviews	Were any interviews repeated? How many?	No.
Audio/video recordings	Did the research use audio or video recordings to collect data?	Yes. For face-to-face interviews, a voice recorder was used with prior and verbal consent. For online interviews, the app’s recording tool was used.
Field notes	Were field notes made during and/or after the interviews or focus groups?	No field notes were made during interviews. Memos were created during analysis in Atlas.ti.
Duration	What was the duration of the interviews or focus groups?	Interviews lasted between 40 min and 1 h.
Data saturation	Was data saturation discussed?	No. Due to the small sample size, data saturation was not addressed.
Transcript return	Were transcripts returned to participants for comments and/or corrections?	No. Transcripts were not returned, but their integrity was ensured in the informed consent.
Domain 3: Data analysis and findings		
Data analysis		
Number of data coders	How many coders analyzed the data?	N = 1.
Coding tree description	Did the authors provide a description of the coding tree?	Yes. Two coding trees were provided: one showed code relationships (Figure 1) and the other showed co-occurrence strength (Supplementary Materials).
Theme identification	Were themes identified in advance or derived from the data?	Themes were derived from the data by grouping codes from interviews.
Software	What software was used to manage the data?	ATLAS.ti Mac (version 24.2.0).
Participant checking	Did participants provide feedback on the findings?	No. Feedback has not yet been provided but will be.
Presentation of findings		
Quotations	Were participant quotations presented to illustrate themes/results? Were they identified in the text?	Yes. Verbatim quotes were presented and identified by group and interview number.
Data and findings consistency	Was there consistency between the data and the findings?	Yes.
Clarity of major themes	Were major themes clearly presented in the results?	Yes.
Clarity of minor themes	Is there a description of diverse cases or discussion of minor themes?	Yes.

### Appendix B.2. JBI Critical Appraisal Checklist for Qualitative Research

**Item**	**Yes**	**No**	**Unclear**	**Not Applicable**
1. Is there congruity between the stated philosophical perspective and the research methodology?	X			
2. Is there congruity between the research methodology and the research question or objectives?	X			
3. Is there congruity between the research methodology and the methods used to collect data?	X			
4. Is there congruity between the research methodology and the representation and analysis of data?	X			
5. Is there congruity between the research methodology and the interpretation of results?	X			
6. Is there a statement locating the researcher culturally or theoretically?		X		
7. Is the influence of the researcher on the research, and vice versa, addressed?		X		
8. Are participants, and their voices, adequately represented?	X			
9. Is the research ethical according to current criteria, or for recent studies, and is there evidence of ethical approval by an appropriate body?	X			
10. Do the conclusions drawn in the research report flow from the analysis or interpretation of the data?	X			
