# Supplementary figures and images for: Analysis of Determinant Factors and Mechanisms in Early Childhood Care Services: A Qualitative Study in the Asturian Context (Spain)
Source: Children (Basel). 2025 Aug 17;12(8):1079. doi: 10.3390/children12081079 (PMC12385120; doi:10.3390/children12081079)

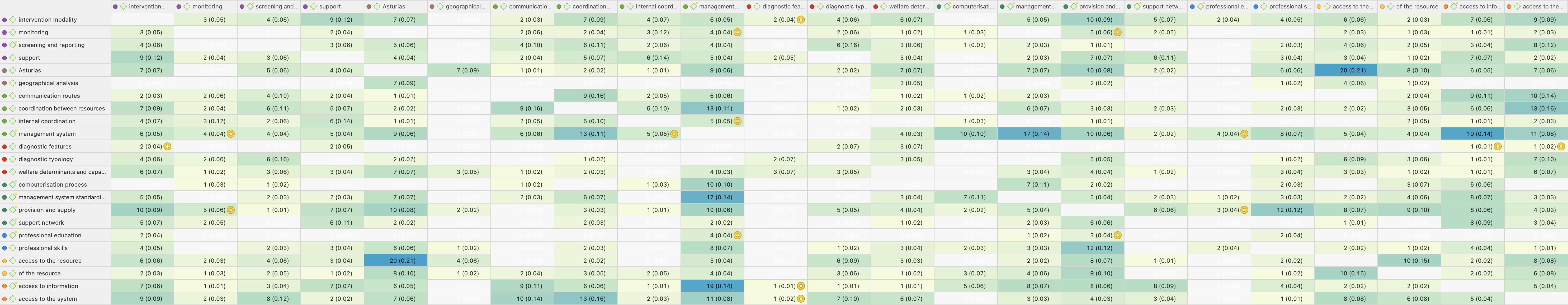

Supplement: Supplementary file 1 [file children-12-01079-s001.zip › children-3752112-supplementary.png]
